# The use of histopathological subtyping in patients with ampullary cancer: a nationwide analysis

**DOI:** 10.1186/s12957-022-02873-y

**Published:** 2022-12-24

**Authors:** Jacob de Bakker, Dirkje Sommeijer, Marc Besselink, Geert Kazemier, Nicole van Grieken

**Affiliations:** 1 Department of Surgery, Amsterdam UMC, Vrije Universiteit, Amsterdam, The Netherlands; 2 Cancer Center Amsterdam, Amsterdam, The Netherlands; 3 Department of Medical Oncology, Amsterdam UMC, University of Amsterdam, Amsterdam, The Netherlands; 4 Department of Surgery, Amsterdam UMC, University of Amsterdam, Amsterdam, The Netherlands; 5 Department of Pathology, Amsterdam UMC, Vrije Universiteit, Amsterdam, The Netherlands

**Keywords:** Ampullary cancer, Histopathological subtype, Intestinal type adenocarcinoma, Pancreatobiliary adenocarcinoma, Immunohistochemistry

## Abstract

**Background:**

Recent guidelines advise to subtype adenocarcinoma at the ampulla and papilla of Vater (here: ampullary cancer) as intestinal, pancreatobiliary, and mixed, because this has consequences for both prognosis and treatment. This nationwide study aimed to investigate how often histopathological subtyping is performed in daily clinical practice in patients with ampullary cancer.

**Methods:**

Pathology reports of all patients with ampullary cancer were retrieved from the Dutch nationwide pathology database (PALGA, 1991-2020). Reports were assessed for the presence and methods used for the classification of these tumors into intestinal, pancreatobiliary, and mixed subtypes. The use of immunohistochemical markers was recorded.

**Results:**

Overall, 5246 patients with ampullary cancer were included. In 1030 (19.6%) patients, a distinction between intestinal, pancreatobiliary, and mixed subtypes was made. Use of subtyping increased from 3% in 1991–1993 to 37% in 2018–2020. In 274 of the 1030 (26.6%) patients, immunohistochemistry was used to make this distinction. A gradual increase in the use of various immunohistochemical markers was seen over time since 2008, with cytokeratin 7, cytokeratin 20, and CDX2 being the most common. Staining of DPC4/SMAD4 was increasingly used since 2012.

**Conclusion:**

Despite recent improvements in the use of subtyping in ampullary cancer, the distinction between intestinal, pancreatobiliary, and mixed subtypes is only made in a minority of patients. Nationwide efforts are required to standardize the pathological distinction of the various subtypes of ampullary cancer.

**Supplementary Information:**

The online version contains supplementary material available at 10.1186/s12957-022-02873-y.

## Introduction

Adenocarcinoma originating at the ampulla and papilla of Vater (here: ampullary cancer) includes the intestinal and pancreatobiliary subtypes which are associated with different long-term survival [1, 2]. It has been suggested to occur slightly more frequent in male patients, with a wide age range at diagnosis. Although the overall incidence in Western countries is less than 0.5 cases per 100,000 individuals according to data from international registries, the incidence rate has shown a significant increase over the last decades [3].

Patients with the intestinal subtype have a significantly longer overall survival compared to patients with the pancreatobiliary subtype [4–6]. Patients with intestinal subtype showed similar outcomes as those with duodenal adenocarcinoma. Since intestinal subtype and duodenal adenocarcinoma originate from the same epithelial cell type, with comparable tumor behavior, this provides an obvious rationale for similar systemic and local treatment [4, 6].

Adjuvant chemotherapy is advised in patients with resected pancreatic ductal adenocarcinoma in order to improve overall survival [7, 8]. Some studies have observed that patients with pancreatobiliary subtype, mimicking pancreatic ductal adenocarcinoma, may also benefit from adjuvant systemic therapy, improving overall survival, whereas intestinal subtype does not benefit from this same regimen [9, 10]. On the other hand, local treatment of oligometastases of the intestinal subtype may improve the survival of patients with the intestinal subtype [11], whereas in advanced stage of pancreatobiliary subtype only gemcitabine has been shown to be effective [12, 13].

The distinction between pancreatobiliary and intestinal subtypes impacts prognosis and treatment decisions. Therefore, this study aims to investigate how often a pathological distinction was made between the intestinal subtype and the pancreatobiliary subtype at the ampulla of Vater in The Netherlands, how this distinction was made, and how this has developed over the years.

## Methods

### Histopathology database

In The Netherlands, all histopathology and cytopathology reports are collected in a national archive (*Pathologisch Anatomisch Landelijk Geautomatiseerd Archief* or PALGA), which encompasses all 64 pathology laboratories nationwide. Between 1971 and 1991, an increasing number of laboratories joined PALGA. Since 1991, PALGA has achieved nationwide coverage and currently contains 42 million pathology reports from nearly 10 million patients [14]. Every pathology report in PALGA contains encrypted patient identification, part of the summary of the original pathology report and diagnostic codes similar to the Systematised Nomenclature of Medicine (SNOMED) issued by the College of American Pathologists [15]. Each pathology report can, however, be traced back to individual (but anonymized) patients with a unique identifier, allowing follow-up of subsequent histology or cytology, irrespective of the hospital where subsequent specimens for pathological evaluation are obtained [16]. The diagnostic code contains a term indicating the anatomical location, type of specimen, and a morphological term describing the diagnosis. For each pathology report, sex, age, date of the pathological evaluation, detailed microscopic report, conclusion text, and diagnostic codes were made available. PALGA does not contain full information on disease stage in all patients included in this nationwide database. The present study was based on data recorded in the PALGA database between 1991 and 2020.

### Patients

For the present study, all patients registered in the PALGA database between 1991 and 2020 with an initial, histologically proven ampullary cancer were identified. The search codes that were used to identify these patients are described in the Appendix (A1). For each patient in the cohort, all pathology reports from either biopsies, surgical resections, or both were retrieved. All pathology reports were scrutinized for codes implicating a different type of cancer and for duplicates. Duplicates were pathology reports which were found twice in the database, e.g., a double report stating the same pathology report. Subclassification of adenocarcinomas into a mucinous type of signet ring cell type adenocarcinoma was not considered to be distinctive for the pancreatobiliary or intestinal type adenocarcinoma. If a distinction between the intestinal subtype and the pancreatobiliary subtype at the ampulla of Vater region was made, the methods used were investigated.

### Statistical analysis

Frequencies were calculated and presented in tables and figures using IBM SPSS 23.0 (IBM SPSS, Chicago, IL).

## Results

### Study cohort

**Fig. 1:**
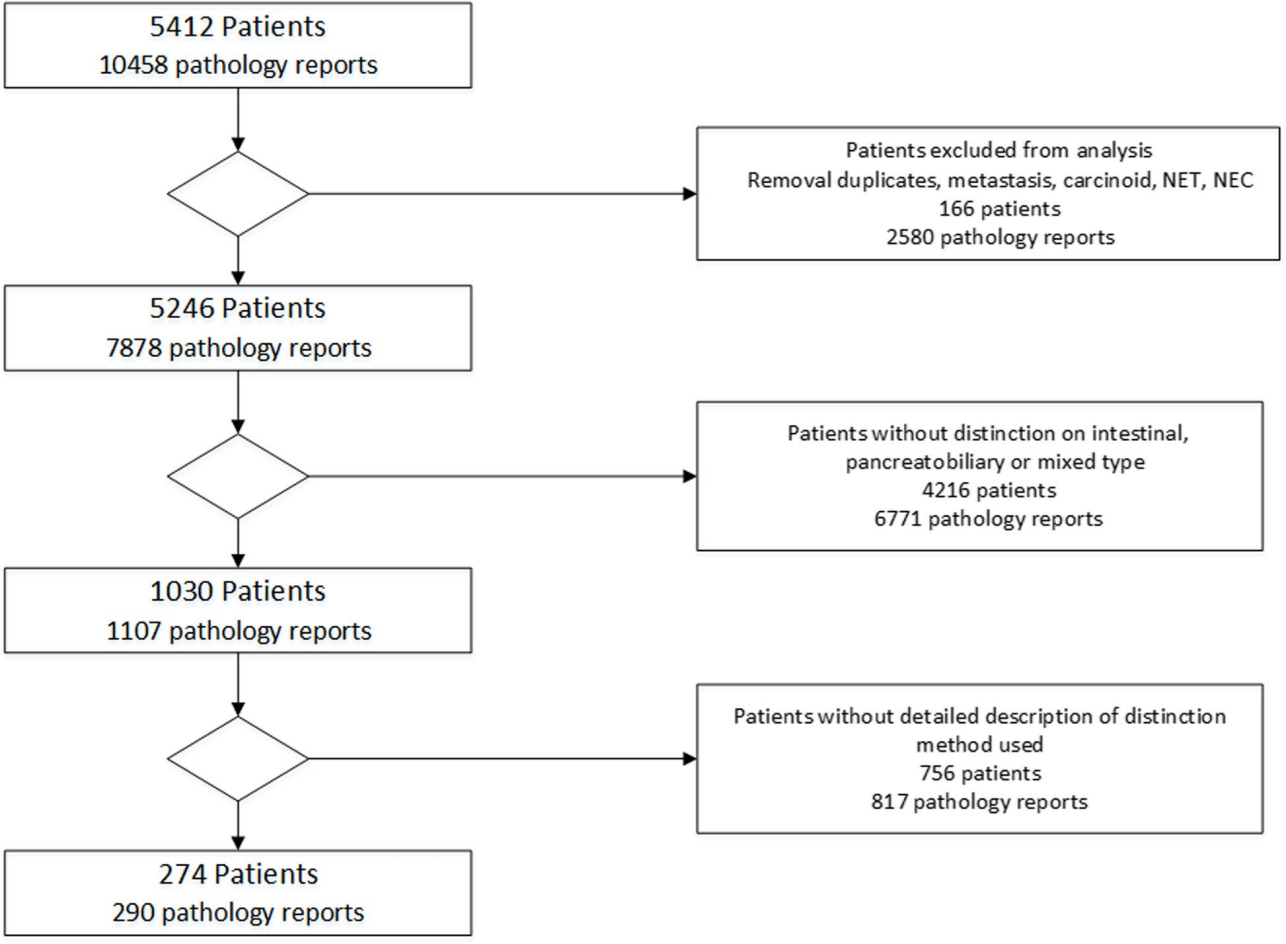
Study cohort selection

Overall, 5412 patients with ampullary cancer were included. Of these, 166 patients were excluded based on an erroneous diagnostic code or metastatic disease located at the ampulla of Vater, leaving a total of 5246 patients for further analysis (Fig. 1).

A distinction between pancreatobiliary subtype, intestinal subtype, and mixed type adenocarcinoma was made in 1030 (19.6%) out of the 5246 patients. This distinction was made either on the biopsy, the resection specimen, or both. A higher number of pathology reports than patients was found due to the fact that patients could have more than one pathology report caused by multiple pathological investigations, e.g., biopsy followed by resection specimen, or the same specimen was reviewed in another center. In the cohort of 1030 patients (1107 pathology reports) in five patients, a discordance was found between the pathology reports. In four of these five patients, the tumor was of the intestinal subtype on the biopsy, while in the resection specimen it appeared to be of the pancreatobiliary subtype.

Table 1 shows the type of specimen and its pathological results from patients (*N*=1030) in whom a distinction was made. Supplementary table 1 shows the type of histological examinations performed and the distinction made based on all pathology reports (*N*=1107) Again, the number of pathology reports is higher than the number of patients because some patients had more than one pathology report (e.g., biopsy and resection).

**Table 1:** Type of histological examinations and its pathological results from a patient perspective (duplicates removed)

	Pancreatobiliary type	Intestinal type	Mixed type	Total (%)
Biopsy	43 (39%)	68 (61%)	0	111 (11%)
Resection	522 (58%)	356 (40%)	21 (2%)	899 (87%)
Revision biopsy	3 (33%)	6 (67%)	0	9 (1%)
Revision Resection	7 (67%)	4 (33%)	0	11 (1%)
Total	576 (56)	434 (42%)	21 (2%)	1030 (100%)

### Immunohistochemistry

In 274 of the 1030 (27%) patients where a distinction between pancreatobiliary and intestinal subtype was reported, in a total of 295 pathology reports, immunohistochemistry was used to make this distinction. In all other patients where a distinction was made, details about the method were lacking. A detailed description of the immunohistochemical evaluation was reported in 290 of 295 pathology reports. In 16 out of 274 patients, two pathology reports were included in this analysis on the use of immunohistochemistry.

**Fig. 2:**
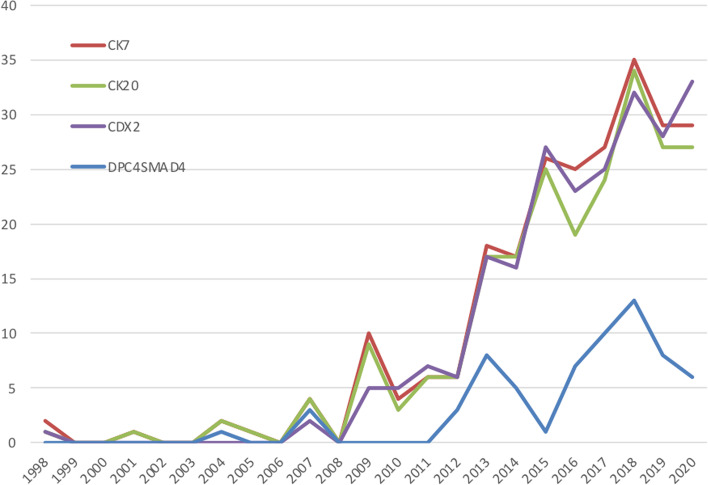
Overview of the most commonly used immunohistochemical markers in patients with ampullary cancer over time

A gradual increase in the use of various immunohistochemical markers is seen over time, starting around 2008, whereas DPC4/SMAD4 is being commonly used since 2012 (Fig. 2). Before, these markers were used only sporadically. Most commonly used markers were cytokeratin 7 (CK7), cytokeratin 20 (CK20), and CDX2 in 242, 223, and 227 reports, respectively. In 189 pathology reports, these markers were used in various combinations.

Tumors that were CK7 positive (CK7+), CK20 negative (CK20−), and CDX2 negative (CDX2−) were classified as pancreatobiliary subtype in 53 of 57 (93%) cases compared to mixed type (*n*=0) and intestinal subtype (*n*=4). Tumors that were CK7−, CK20+, and CDX2+ were classified as an intestinal subtype in 31 of 34 (91%) cases, compared to mixed type (*n*=0) and pancreatobiliary subtype (*n*=3). The combination CK7−, CK20+, and CDX2− was found in only one report in which the conclusion was intestinal subtype. The combination CK7−, CK20−, and CDX2− was found in two reports, both classified as pancreatobiliary subtype.

In 83 of 189 pathology reports, CK7, CK20, and CDX2 were used in any combination with immunohistochemistry for at least one mucine, including MUC1 (*n* = 46), MUC2 (*n* = 23) and/or MUC5 (*n* = 4). MUC1 was more commonly described in the pancreatobiliary subtype, *n* = 33 (72%), and MUC2, *n*=11 (48%), was more frequently shown in the intestinal subtype. MUC5 was only described in 27 pathology reports.

In 59 pathology reports, DPC4/SMAD4 was used. Tumors that were DPC4/SMAD4 positive were classified as pancreatobiliary subtype in 24 of 42 (57%) cases compared to mixed type (*n* = 2) and intestinal subtype (*n* = 16). Tumors that were DPC4/SMAD4 negative were classified as pancreatobiliary subtype in 12 of 17 (71%) cases compared to mixed type (*n* = 1) and intestinal subtype (*n* = 4). DPC4/SMAD4 has more often been used in combination with CK7, CK20, and CDX2 (*n* = 34) compared to MUC1, MUC2, and MUC5 (*n* = 15).

### Trends over time

**Fig. 3:**
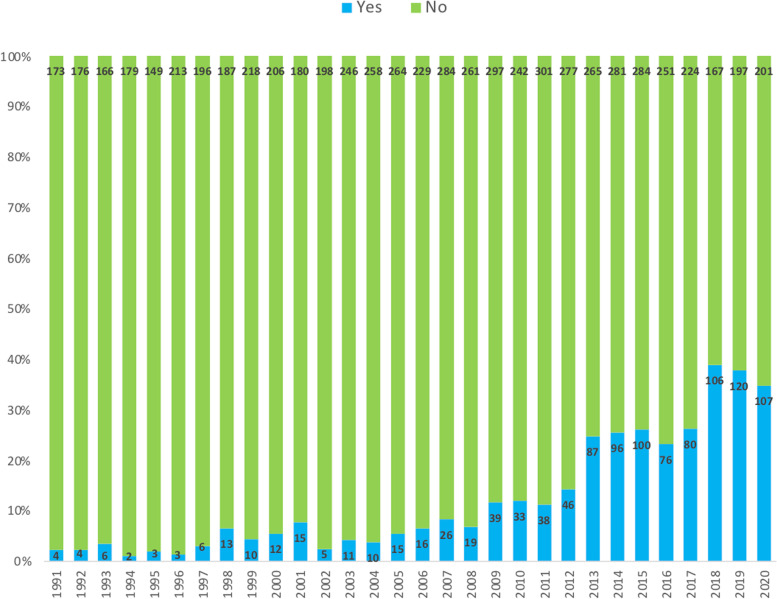
Distinction made between pancreatobiliary and intestinal type ampullary cancer. Distinction between pancreatobiliary and intestinal type adenocarcinoma and its development in time. All pathology reports in which distinction was made in pancreatobiliary, intestinal, or mixed types are combined as Yes

Figure 3 shows the frequency in which a histopathological distinction in subtypes was made over time. This varied from 2% in 1991 to 35% in 2020.

## Discussion

This first nationwide analysis on the use of histopathological subtyping in patients with ampullary cancer found that this distinction was only made in 19.6% of patients over a period of four decades. Even though an increase was observed, still in the last three study years only in 37% of patients subtyping was performed. When reported (27%), the distinction was most commonly based on immunohistochemical analysis using a combination for CK7, CK20, and CDX2.

The exact contribution of morphology and immunohistochemistry by the pathologist is however not reported in many cases in our study cohort. For example, CK7+, CK20−, and CDX2− are mostly classified as pancreatobiliary, but sometimes as intestinal as well. In this particular example, the pathologist may have placed more value on morphological characteristics, especially if CK7 showed only weak staining intensity. Subtyping DPC4/SMAD4 tumors into the intestinal subtype or pancreatobiliary is probably the result of combining DPC4/SMAD4 staining with other markers like CK7, CK20, and CDX2. Unfortunately, the data do not allow for a detailed analysis of this point.

Several studies reported a difference in survival between patients with intestinal and pancreatobiliary subtypes of ampullary cancer [5, 6]. A retrospective single-center study in 966 patients showed a median survival of 23 months for pancreatobiliary subtype and of 71 months for the intestinal subtype after curative intent resection. Neoadjuvant or adjuvant treatment was not taken into consideration [4]. A retrospective single-center study in 104 patients showed significantly worse 5-year disease-free survival in patients with pancreatobiliary versus intestinal subtype (47.8% versus 73.1%) [5]. In that study, the use of adjuvant chemoradiotherapy did not influence survival rates significantly. These prognostic differences are considered a reflection of differences in biological behavior of these tumor subtypes and substantiate that pancreatobiliary subtype and intestinal subtype are distinct entities.

Positive IHC staining on CK20 or CDX2 is more common in the intestinal subtype and negative staining on CK20 or CDX2 is more seen in the pancreatobiliary subtype. On the other hand, CK7 is more often expressed by the pancreatobiliary subtype [17]. A recent study in 30 patients, identified CK7 and CDX2 as the 2 most discriminating immunohistochemical markers for the distinction between pancreatobiliary subtype and intestinal subtype [18]. Mucins are high-molecular-weight glycoproteins expressed by epithelial tissues. They have a high content of clustered oligosaccharides, forming a mucosal protection layer at the surface of the gastrointestinal lining [19]. In concordance with the literature, the present study shows that MUC1 was more commonly found in the pancreatobiliary subtype and MUC2 was more frequently found in the intestinal subtype [20–22].

SMAD4 serves as the central mediator of transforming growth factor β (TGF-β) signaling. It is specifically inactivated in over half of pancreatic ductal adenocarcinoma, and varying degrees in many other types of cancers. It is an independent predictor of poor prognosis in pancreatic ductal adenocarcinoma [23]. In a single-center study in Denmark, SMAD4 was found to be one of the most frequent genetic alterations in 20.4% of 59 cases. This cohort consisted of surgical specimens collected between 2010 and 2018 [24].

Surgical resection is the cornerstone of the treatment of early-stage ampullary cancer. Systemic therapy is used in all stages of ampullary cancer. This could include neoadjuvant and adjuvant therapy for localized, potentially resectable disease, and first-line or subsequent therapy for locally advanced, metastatic, and recurrent disease [25]. There is evidence to suggest that the pancreatobiliary subtype and intestinal subtype differ in their response to adjuvant chemotherapy regimens [9, 10, 25]. Also in the metastatic setting, there is some evidence that the subtyping of ampullary adenocarcinoma is useful to determine the type of palliative chemotherapy. Until data from randomized trials become available, it seems reasonable to treat the pancreatobiliary subtype and intestinal subtype similar to the type of cancer with whom they share their cell type of origin [26, 27].

Histopathological subtyping may direct clinicians towards the most effective type of treatment. For example, upfront resection of oligometastatic disease may be of potential benefit specifically in patients with intestinal subtype. A previous small pilot study showed a survival benefit after resection of oligometastasis in duodenal adenocarcinoma [11]. In a retrospective single-center study including 155 patients with duodenal cancer, a median overall survival of 37 months for patients receiving local treatment of metastases versus 14 months for patients receiving systemic treatment only [11]. Since the biological behavior of the pancreatobiliary subtype is more aggressive, there is little benefit to be expected from the local treatment of oligometastatic disease, comparable to metastastic pancreatic cancer These considerations again emphasize the importance of distinguishing the pancreatobiliary subtype from the intestinal subtype.

The present data show a substantial increase in the percentage of patients in which the pancreatobiliary subtype and intestinal subtype were distinguished since 2012 up to 38.9% in 2018 (Fig. 3). In the most recent decade, standardized structured reporting for pathology reporting of resection specimens of both pancreatic and ampullary carcinomas was implemented in the Netherlands which requires subtyping of ampullary carcinomas, which may have raised awareness among pathologists to distinguish the pancreatobiliary subtype from the intestinal subtype. Clearly, this proforma requires further implementation.

The findings reported in this study should be interpreted in light of several limitations. First, the lack of a detailed description of the intensity in staining of the immunohistochemical markers used to make a distinction between the pancreatobiliary subtype and the intestinal subtype. Expression of markers is often not a black or white situation. CK7 is usually strongly expressed in the pancreatobiliary subtype, but could be, although often weaker expressed in the intestinal subtype. Similarly, CDX2 is associated with the intestinal subtype, but can also show some expression in the pancreatobiliary subtype. Second, heterogeneous expression may be seen in various tumor areas. These potential variations are difficult to extract or quantify from pathology reports. Even if these data were available, no consensus how to interpret these stainings is currently available. Ang et al., Kumari et al., and Sree et al. reported a threshold of 10% to determine if a staining is positive [9, 17, 28, 29], whereas Bakshi et al. proposed 25% as a cut-off value to determine a marker to be positive [9]. Bronsert et al. used a cut-off of 5% with regard to positive tumor staining [4]. As an alternative, Chu et al. divided staining patterns in focally positive (5–50%) and diffusely positive (>50%) [30]. As long as no clear definitions are available, it is at the pathologists’ discretion how to interpret immunohistochemical staining patterns. Third, the lack of knowledge on the disease stage in all included patients as PALGA does not provide such information. Statistical analyses involving disease stage could therefore not be performed.

In conclusion, the distinction between the intestinal and pancreatobiliary subtypes has only been made in a minority of patients with ampullary cancer over the last four decades in the Netherlands. Despite some improvement, in the most recent 3 years, this distinction was only made in 37% of patients. Because of the profound difference in clinical biology, pathologists are encouraged to distinguish the intestinal subtype from the pancreatobiliary subtype in all future patients suffering from ampullary cancer since this has important clinical implications.

### Supplementary Information

**Additional file 1:** **Supplementary Table 1.** Type of histological examinations performed and the distinction made based on all pathology reports.

## Data Availability

The dataset supporting the conclusions of this article is fully available upon request.

## Appendix

The following SNOMED-like codes were used:

PALGA diagnosis codes used in the analysis:

Papilla of Vater: T58700

Different type of adenocarcinomas: M8140, M8260, M8163, M8144, M8480, M8440, M8262, M8145, M8576, M8470, M8450, M8141, M8441, M8213, M8261

PALGA terminology: https://www.palga.nl/palga-on-line-thesaurus.html
