# Supplementary material for: The use of histopathological subtyping in patients with ampullary cancer: a nationwide analysis
Source: World J Surg Oncol. 2022 Dec 24;20:406. doi: 10.1186/s12957-022-02873-y (PMC9789567; doi:10.1186/s12957-022-02873-y)
Supplement: Supplementary file 1 — Additional file 1: Supplementary Table 1. Type of histological examinations performed and the distinction made based on all pathology reports. [file 12957_2022_2873_MOESM1_ESM.docx]

**Supplementary Table 1**

|  | Pancreaticobiliary type | Intestinal type | Mixed type | Total Pathology reports (%) |
| --- | --- | --- | --- | --- |
| Biopsy | 44 (37%) | 74 (63%) | 0 | 118 (11%) |
| Resection | 538 (58%) | 373 (40%) | 22(2%) | 933 (84%) |
| Revision Biopsy | 9 (43%) | 12 (57%) | 0 | 21 (2%) |
| Revision Resection | 19 (54%) | 14 (40%) | 2 (6) | 35(3%) |
| Total | 611 (55%) | 473 (43%) | 24 (2%) | 1107 (100%) |

Type of histological examinations performed and the distinction made based on all pathology reports
